# Identification of Amino Acid Residues in Human IgM Fc Receptor (FcµR) Critical for IgM Binding

**DOI:** 10.3389/fimmu.2020.618327

**Published:** 2021-01-27

**Authors:** Christopher M. Skopnik, Khlowd Al-Qaisi, Rosaleen A. Calvert, Philipp Enghard, Andreas Radbruch, Brian J. Sutton, Hiromi Kubagawa

**Affiliations:** ^1^ Humoral Immune Regulation, Deutsches Rheuma-Forschungszentrum, Berlin, Germany; ^2^ Randall Centre for Cell and Molecular Biophysics, King’s College London, London, United Kingdom; ^3^ Department of Nephrology and Medical Intensive Care, Charité-Universitätmedizin, Berlin, Germany

**Keywords:** IgM, Fc receptor, Fcμ receptor, polymeric ig receptor, FcμR

## Abstract

Both non-immune “natural” and antigen-induced “immune” IgM are important for protection against infections and for regulation of immune responses to self-antigens. The roles of its Fc receptor (FcµR) in these IgM effector functions have begun to be explored. In the present study, by taking advantage of the difference in IgM-ligand binding of FcµRs of human (constitutive binding) and mouse (transient binding), we replaced non-conserved amino acid residues of human FcµR with mouse equivalents before establishment of cell lines stably expressing mutant or wild-type (WT) receptors. The resultant eight-different mutant FcµR-bearing cells were compared with WT receptor-bearing cells for cell-surface expression and IgM-binding by flow cytometric assessments using receptor-specific mAbs and IgM paraproteins as ligands. Three sites Asn66, Lys79-Arg83, and Asn109, which are likely in the CDR2, DE loop and CDR3 of the human FcµR Ig-like domain, respectively, were responsible for constitutive IgM binding. Intriguingly, substitution of Glu41 and Met42 in the presumed CDR1 with the corresponding mouse residues Gln and Leu, either single or more prominently in combination, enhanced both the receptor expression and IgM binding. A four-aa stretch of Lys24-Gly27 in the predicted A ß-strand of human FcµR appeared to be essential for maintenance of its proper receptor conformation on plasma membranes because of reduction of both receptor expression and IgM-binding potential when these were mutated. Results from a computational structural modeling analysis were consistent with these mutational data and identified a possible mode of binding of FcµR with IgM involving the loops including Asn66, Arg83 and Asn109 of FcµR interacting principally with the Cµ4 domain including Gln510 and to a lesser extent Cµ3 domain including Glu398, of human IgM. To our knowledge, this is the first experimental report describing the identification of amino acid residues of human FcµR critical for binding to IgM Fc.

## Introduction

Antibodies or immunoglobulin (Ig) molecules, key players in humoral immunity, have dual binding activities to antigens *via* their N-terminal variable domains in the Fab region and to effector molecules *via* their C-terminal constant domains in the Fc region. One example of the latter is a family of cell surface Fc receptors (FcRs). FcRs for switched Ig isotypes (*i.e.*, IgG, IgE, IgA) are expressed by various hematopoietic cell types, thereby functioning as central mediators coupling innate and adaptive immune responses (1). By contrast, FcR for IgM (FcµR), the newest member of the FcR family (2), is expressed by lymphocytes only: B, T, and NK cells in humans and only B cells in mice, although there are some conflicting data regarding FcµR expression by non-B cells in mice (2–13). By computational analysis of existing genomic sequence databases, the distribution of *FCMR* orthologues seems to be restricted to mammals (14). Given the facts that IgM is the first Ig isotype to appear during phylogeny, ontogeny and immune responses and serves as a first line of defense against pathogens, the lymphocyte-restricted expression of FcµR and its selective distribution in mammals are unexpected. This may in turn suggest that FcµR must possess a function distinct from FcRs for switched Ig isotypes (12).

Both human and mouse FcµR genes are located in a syntenic region of chromosome 1 adjacent to two other IgM-binding receptor genes, one encoding the polymeric Ig receptor (pIgR) and the other the FcR for both polymeric IgA and IgM (Fcα/µR) (2, 3, 6). While the ligand-binding domains of these three receptors are similar to each other, FcµR seems to be the most distantly related among them, consistent with their distinct interactions with ligands: FcµR for IgM only *vs* pIgR and Fcα/µR for both polymeric IgA and IgM (2, 6). In our previous studies with transductants stably expressing FcµR cDNAs, human receptor-bearing cells exhibited IgM-ligand binding irrespective of the growth phase in culture (*constitutive* binding), whereas mouse receptor-bearing cells bound to IgM during the pre-exponential phase only (*transient* binding), despite no significant changes during cell culture in the surface FcµR levels as determined by receptor-specific mAbs (6). Subsequent domain swapping analyses between human and mouse FcµRs revealed that the distinct ligand-binding activity observed with these two receptors was directly attributed to their Ig-like domains responsible for IgM binding rather than indirectly to other parts of the receptors (6). In the present study, we have defined the amino acid (aa) residues involved in the constitutive ligand-binding of human FcµR by site-directed mutagenesis based on the comparison between human and mouse receptors, and modeled the interaction between human FcµR and IgM.

## Materials and Methods

### FcµR Transductants

**Figure 1:**
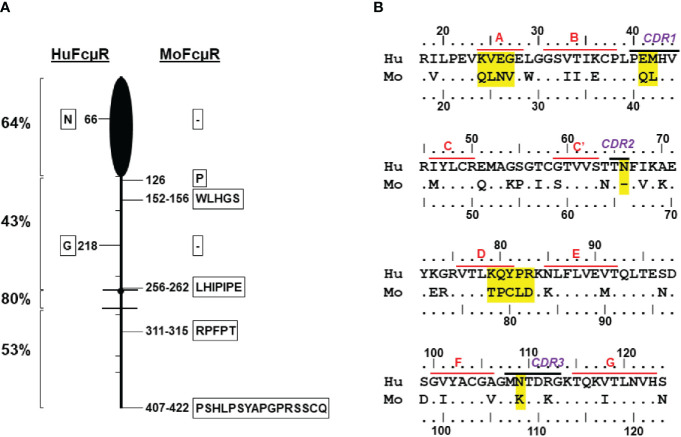
Human *versus* mouse FcµR. **(A)** Schematic presentation of homology between human and mouse FcµRs. FcµR is depicted as a badminton-like shape consisting of amino-terminal Ig-like domain (black closed oval shape), stalk region (above the top line), transmembrane (between the two lines) and the cytoplasmic tail (below the bottom line). Hatch marks indicate exon boundaries and small closed circle in the transmembrane region indicates a charged His residue. Left numbers indicate percent identity in the indicated regions between human and mouse receptors. The positions of aa addition (single letter code within frame) or gap (- within frame) between human (left) and mouse (right) FcµR are shown along the cartoon. **(B)** Amino acid sequence alignment of the Ig-like domains of human and mouse FcµRs. The numbers indicate the aa position from the first M residue of human and mouse FcµR on the top and bottom of the sequence, respectively. Amino acid identity is indicated by dots (•) and gaps by dashes (-). Predicted ß-strands and complementary determining regions (CDRs) of human FcµR are indicated. Accession numbers of human and mouse FcµRs in NCBI are NM_005449 and NM_026976, respectively.

The coding sequence of human FcµR (huFcµR; *FCMR*, NM_005449 in NCBI) and mouse FcµR (moFcµR; *Fcmr*, NM_026976) cDNAs was flanked by the restriction enzyme sites of *Bgl*II (for huFcµR) or *Sac*II (for moFcµR) and of *Cla*I (for both) at the 5’ and 3’ sites, respectively, and was synthesized as a wild type (WT) by Eurofins Genomics (Berlin, Germany). HuFcµR cDNAs encoding the following replacement mutations with the corresponding moFcµR aa residues were similarly synthesized: KVEG24-27QLNV [designated as 24–27 for simplicity], E41Q, M42L, EM41-42QL [41–42], N66-, KQYPR79-83TPCLD [79–83], Y81C, and N109K (see **Figure 1B**). The numbers indicate the aa position from the first M residue of the huFcµR sequence and were used for alignment of the moFcµR aa residues. After verifying the correct sequences with the expected replacements of the resultant cDNAs, *Bgl*II/*Cla*I-cut, WT and mutant huFcµR cDNA inserts and *Sac*II/*Cla*I-cut WT moFcµR cDNA insert were subcloned into the pRetroX-IRES-ZsGreen1 retroviral expression vector (Takara) and transfected into the PLAT-E packaging cell line (kindly provided by Dr. Toshio Kitamura) by Xfect*™* (Takara), before transduction in the BW5147 mouse thymoma line as previously described (2). The pRetroX-IRES-ZsGreen1 vector only (without insert cDNA) was also transduced as an empty vector control. BW5147 cells stably expressing GFP were enriched by fluorescence activating cell sorter (FACS). The first seven N-terminal mutants were prepared together in the first series of experiments and the C-terminal mutant was made in the second series of experiments.

### Immunofluorescence Analysis

For cell surface expression of FcµR, an equal mixture of WT control BW5147 (GFP-negative) cells and GFP-positive transduced cells was first incubated with mouse mAbs specific for huFcµR [clone HM14 (γ1κ isotype; stalk region-specific) and HM7 (γ2bκ; IgM-ligand-binding site-specific)] or moFcµR [MM3 (γ1κ) and MM24 (γ2bκ; both stalk region-specific)] at the predetermined protein concentrations as described (2, 15, 16). For IgM-ligand binding, the similar mixture of cells was incubated with mouse IgMκ paraprotein TEPC183 (Sigma-Aldrich, Darmstadt, Germany) at 3 µg/ml or PBS. In our previous studies (2), mouse IgM bound to huFcµR better than human IgM. In this study, where the mouse IgM was used, results were checked with biotin-labeled, human myeloma IgM, followed by PE-labeled streptavidin. Bound mAbs or IgM ligands were identified by addition of PE-labeled, polyvalent goat antibodies specific for mouse Igs (Southern Biotechnology Associates, Birmingham, AL) as a developer (2, 17). For compensation purposes, PE-labeled mouse anti-Thy1.1 mAb [OX7 (γ1κ)] was included to stain WT BW5147 cells. Stained cells were examined by BD FACSCanto ll flow cytometer along with FACSDiva software (BD Bioscience), and flow cytometric data were analyzed with FlowJo software (Becton Dickinson). The mean fluorescence intensities (MFIs) of PE and GFP in both control and transduced cell populations were assessed for each transductant. The indices of MFIs of each mAb and IgM ligand in transductants were defined as follows: MFI index of a given mAb or IgM ligand in a given transductant/control mixture = [(PE MFI of the transductant) - (PE MFI of the control)] ÷ [(GFP MFI of the transductant) - (GFP MFI of the control)].

### Modeling FcµR Ig-Like Domains

The sequence used for modeling the human FcµR domain (GenBank NP_005440) was: LRILPEVKVEGELGGSVTIKCPLPEMHVRIYLCREMAGSGTCGTVVSTTNFIKAEYKGRVTLKQYPRK-NLFLVEVTQLTESDSGVYACGAGMNTDRGKTQKVTLNVHS and for the mouse FcµR domain (GenBank NP_081252) was: LRVLPEVQLNVEWGGSIIIECPLPQLHVRMYLCRQMAKPGICSTVVSN-TFVKKEYERRVTLTPCLDKKLFLVEMTQLTENDDGIYACGVGMKTDKGKTQKITLNVHN. Swiss-Model (18) using pIgR domain 1 (D1) (PDB 5D4K) as template and I-TASSER (19–21) were used to create models from the above sequences.

### Melting Temperature Simulations

Molecular dynamics heat simulations were used to estimate melting temperatures (Tm). Structures for Ig-like domains of human FcµR of WT and mutants and mouse FcµR of WT were generated using Swiss-Model (18). An Amber MD ffSB force field and Amber16 software were used (22). Hydrogen mass repartitioning (23) enabled the use of a 4 fs time step. A salt concentration of 150 mM was used. Five repeats of each model were simulated for 1 µs. Models were heated rapidly from -273°C to -73°C in 4 ns and then slowly from -73°C to 127°C in 200 ns. Output files (“.nc” and “.prmtop”) were converted (to “.dcd” and “.pdb” format) for analysis with the Bio3D package (24). The melting event was estimated in two ways. Firstly, native contacts were analyzed in Bio3D, and a moving mean of the proportion of such contacts was calculated; the Tm was that at which 80% of native contacts remained. An alternative definition of the Tm was the point at which a moving mean of the calculated R_g_ of the model exceeded 14Å.

### Molecular Docking of the FcµR/IgM-Fc Complex

Part of the structure of human pentameric IgM-Fc in complex with the pIgR D1 (PDB 6KXS) (25) was used, after removing pIgR D1, to dock the human FcµR Ig-like domain modeled by I-TASSER (21); the docking program used was GalaxyTongDock A (26). As a control, pIgR D1 was successfully docked (score 1,110) onto a fragment of 6KXS containing all the experimentally observed contacts. For the FcµR docking, it was also necessary for computational reasons (limit of 1,000 residues) to reduce the size of the IgM-Fc pentamer, and thus chains C and D of 6KXS were used for the docking site, with chains B and E of the adjacent IgM-Fc subunits of the pentamer to provide the structural context. Heavy-chain tailpieces were removed for all chains as these are not required for FcµR binding (27) and J-chain was not included for the same reason (9, 27). The following constraints were used for the docking to chain C: N66, R83, and N109 (in FcµR, this study), and Q510 (IgM-Fc chain C) (27) are interface residues; E41, M42, and Y81 (in FcµR, this study) and no residues from chains B or E are at the interface. Additional filters were that it was sterically possible for two FcµR domains to bind to the homo-dimeric IgM-Fc subunit (27), that the conserved glycosylation in Cµ3 [at N402, assumed similar in location to that observed at N394 in IgE-Fc (28)] would not interfere with binding, and that most contacts should be with Cµ4 (29). The same constraints were used to dock FcµR onto chain D, but using Q510 of IgM-Fc chain D and excluding contact with chains B and E. The highest scoring docked models that satisfied these criteria were then submitted to the Yasara Energy Minimization Service (30) and contacts were inspected using Chimera (31). Location of FcµR domains on other IgM-Fc subunits by alignment of heavy-chains was performed using YASARA (32).

### Statistical Analysis

All data comparisons were performed using two-sided Student’s *t* test and a *P* value of <0.05 was defined as statistically significant.

## Results

### Differences in the Ig-Like Domains Between Human and Mouse FcµRs

Human FcµR cDNA was initially cloned from cDNA libraries of human B-lineage cells by a functional cloning strategy with IgM-ligand binding (2). Its mouse orthologue was then identified by basic local alignment search technique (BLAST) database analysis. Both cDNAs encode a type I membrane protein consisting of a single VH type Ig-like domain responsible for the ligand binding, an additional extracellular region with unknown domain structure (termed the stalk region), a transmembrane segment (TM) and a long cytoplasmic tail (CY). Unique structural characteristics, such as a charged His residue in the TM and several conserved Tyr and Ser residues in the CY tail, are preserved in both species. However, the overall aa identity between the 390-aa human and 422-aa mouse FcµRs was low (~56%). The degree of homology in each domain was, in order: TM (80%) > Ig-like domain (64%) > CY (53%) > stalk (43%) (**Figure 1**). The mouse receptor had insertions of between 1 and 16 aa in the stalk and CY regions, and a single aa deletion in each of the Ig-like and stalk regions (**Figure 1A** and **Supplemental Figure 1**). Another notable distinction was their IgM-ligand binding activity, as assessed by using a BW5147 thymoma cell line stably expressing human or mouse FcµR. HuFcµR-bearing transductants bound IgM irrespective of their growth phase in cell culture (constitutive binding). By contrast, moFcµR-bearing transductants bound IgM during the pre-exponential growth phase (transient binding), although the surface receptor levels as determined by receptor-specific mAbs were not significantly changed during the entire culture period (6).

To explore the molecular basis for this difference (constitutive *vs* transient binding), we initially made constructs encoding a recombinant human and mouse FcµR fusion protein by swapping each functional domain (*i.e.*, Ig-like, stalk and TM/CY) and examined the IgM ligand binding activity of the resultant chimeric FcµRs. The results indicated that the IgM binding differences in the human and mouse receptors were more directly attributed to the Ig-like domain rather than to some indirect influence by other parts of the receptor (6). The aa sequences of the Ig-like domains of both receptors were thus aligned based on the secondary structure of pIgR D1 as determined by crystallography (33). As shown in **Figure 1B**, several differences are localized around the putative ligand-binding complementary determining regions (CDRs). The negatively charged E41 in the huFcµR CDR1 is an uncharged residue Q in the moFcµR (E *vs* Q). The residue at the next position 42 is a similar nonpolar aa (M or L) in both species, but it is unclear how these two consecutive changes (EM *vs* QL) out of the potentially five aa in the FcµR CDR1 affect their ligand binding activity. Likewise, the N66 in the huFcµR CDR2 is missing in the moFcµR CDR2 (N *vs* -). Another N109 in the huFcµR CDR3 is a positively charged residue of K in the moFcµR (N *vs* K).

In addition to these differences in CDRs, the four aa residues at positions 24–27 in the A-strand of FcµR (see modeling below) are significantly different from each other (KVEG *vs* QLNV). The positively charged K24 and the negatively charged E26 in huFcµR are both uncharged Q and N in moFcµR, respectively (K *vs* Q, E *vs* N). Another distinguishing stretch of residues is at positions 79–83 (KQYPR *vs* TPCLD). These include (*i*) removal of two positively charged residues of K79 and R83 from huFcµR to uncharged T and negatively charged D (K *vs* T, R *vs* D), (*ii*) different positions of P residues at 82 *vs* 80, and (*iii*) at position 81 an aromatic Y in huFcµR *vs* a sulfur-containing C in moFcµR (Y *vs* C). These residues 79–83 correspond to the DE loop of FcµR, directly adjacent to CDR2 (see modeling below). These sequence comparisons led to the hypothesis that if the above different aa residues in human FcµR are replaced by the corresponding mouse aa residues, then the resultant huFcµR mutants may no longer constitutively bind IgM ligands, similar to mouse FcµR.

### Involvement of Three Sites, N66, K79-R83, and N109, for the Constitutive IgM-Binding of Human FcµR

**Figure 2:**
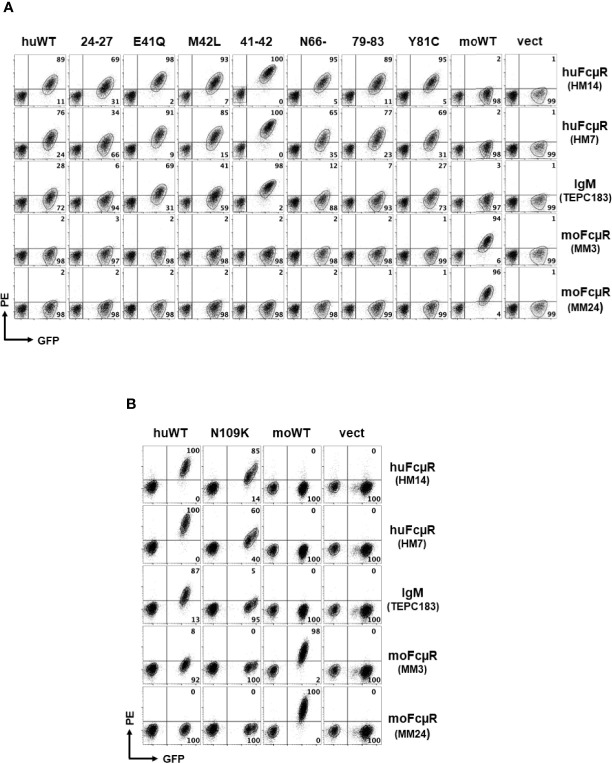
Representative profiles of receptor expression and IgM binding by cells with wild or mutant type of human FcµR. An equal mixture of GFP-negative control cells and GFP-positive cells transduced with the indicated cDNAs (*top label*) was incubated with the indicated FcµR-specific mAbs or a mouse IgM paraprotein (*right label*), before developing with PE-labeled goat anti-mouse Ig antibodies. The stained cells were analyzed by FACSCanto ll. HM14 (γ1κ) and HM7 (γ2bκ) mAbs are specific for an epitope in the stalk region or the ligand binding site of human FcµR, whereas MM3 (γ1κ) and MM24 (γ2bκ) mAbs are specific for an epitope in the stalk region of mouse FcµR. **(A)** Cells transduced with cDNA encoding human FcµR of wild (huWT) or mutant type [KVEG24-27QLNV (24–27), E41Q, M42L, EM41-42QL (41–42), N66-, KQYPR79-83TPCLD (79–83), and Y81C], mouse FcµR (moWT) or vector only (vect) are shown. **(B)** Cells transduced with human FcµR N109K mutant are shown along with huWT, moWT and vect controls. Numbers in A and B indicate % cells in each quadrant among the indicated GFP^+^ cell population.

To test the above hypothesis, we made eight different mutant constructs of huFcµR by replacing the potentially critical residues in its ligand binding domain with the corresponding mouse aa. They included: KVEG24-27QNLV [24–27], E41Q, M42L, EM41-42QL [41–42], N66-, KQYPR79-83TPCLD [79–83], Y81C, and N109K. Human or mouse FcµR of WT and empty vector constructs were also included as controls. These cDNAs were subcloned into a bicistronic retroviral vector with GFP cDNA, packaged, and transduced in the FcµR-negative AKR-derived thymoma cell line BW5147. After enrichment of cells stably expressing GFP by FACS, an equal mixture of GFP-positive transductant and GFP-negative control BW5147 cells was simultaneously assessed by flow cytometry for surface FcµR expression with receptor-specific mAbs and for IgM ligand binding activity. **Figure 2** shows representative profiles in seven N-terminal mutants (**Figure 2A**) and C-terminal mutant (**Figure 2B**) as determined by two different mAbs [HM14 (γ1κ isotype) specific for an epitope in the stalk region and HM7 (γ2bκ) specific for an epitope near the IgM-binding site] and the mouse IgMκ paraprotein TEPC183. Two mAbs specific for moFcµR [MM3 (γ1κ) and MM24 (γ2bκ)] were also included as isotype-matched control mAbs. Both HM14 and HM7 mAbs were clearly but variably reactive with GFP-positive cell populations of WT and mutant huFcµR transductants as compared with GFP-negative control cells. These mAbs did not react with the moFcµR transductant nor with vector-only transductant, thereby confirming their antigen-binding specificity. Conversely, both MM3 and MM24 mAbs specific for moFcµR reacted with GFP-positive moFcµR transductant, but not with others. As expected, reactivity with receptor-specific mAbs was clearly more sensitive for the detection of FcµR than ligand binding with IgM paraproteins (2). MoFcµR cells exhibited minimal binding to IgM as compared with huFcµR WT and mutants.

**Figure 3:**
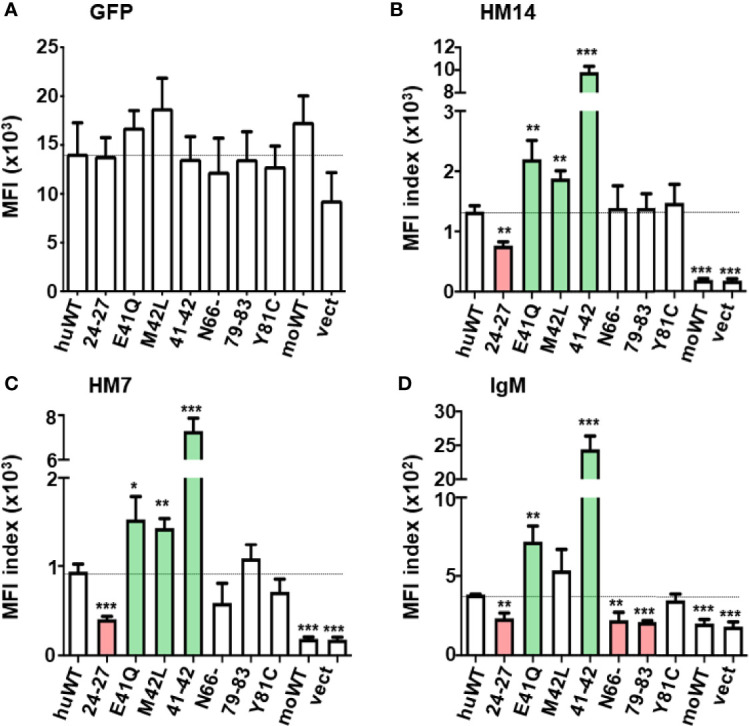
Quantitative assessments of the GFP and FcµR expression and the IgM binding activity by seven N-terminal FcµR mutants. MFI indices of the expression of GFP **(A)**, the reactivity of HM14 **(B)** and HM7 **(C)** and the binding of IgM **(D)** in each transductant were plotted as mean ± 1 SD from three independent experiments described in Materials and Methods. Lines correspond with the MFI index of huWT transductant. *P < 0.05, **P < 0.01, ***P < 0.001 when compared to the huWT transductant.

**Figure 4:**
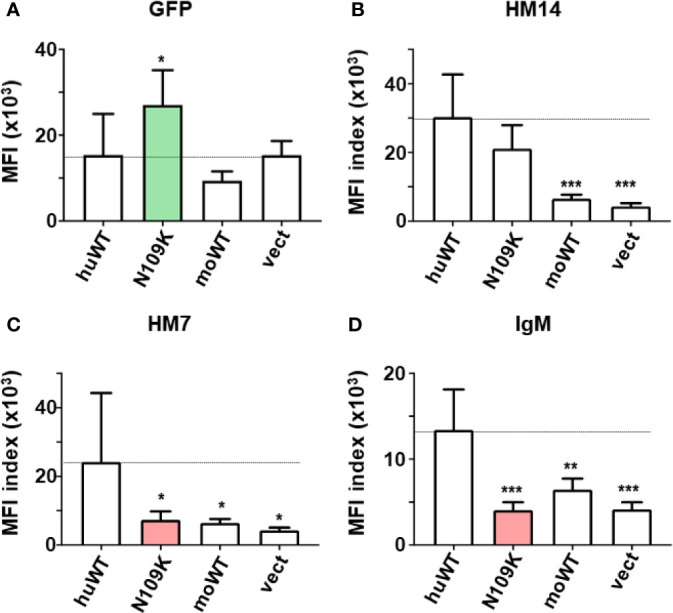
Quantitative assessments of the GFP and FcµR expression and the IgM binding activity by the C-terminal FcµR mutant N109K. MFI indices of the expression of GFP **(A)**, the reactivity of HM14 **(B)** and HM7 **(C)** and the binding of IgM **(D)** by the human FcµR transductants of WT and the N109K mutant as well as in control transductants were plotted as mean ± 1 SD from seven independent experiments. Lines correspond with the MFI index of huWT transductant. *P < 0.05, **P < 0.01, ***P < 0.001 when compared with huWT transductant.

To quantitatively assess the surface receptor density and the ligand binding activity of these transductants, the MFI indices of the GFP expression, the reactivity with receptor-specific mAbs and the IgM-ligand binding activity for each transductant were plotted as mean ± 1 SD from 3 to 7 independent experiments (**Figures 3**, **4**). In the first series of experiments (n = 3) with seven N-terminal mutants, GFP MFIs were comparable among WT and mutant huFcµRs, moFcµR and vector-only transductants (**Figure 3A**). (Although the GFP MFI of vector-only appears to be less than others, this difference was not statistically significant.) The cell surface FcµR levels defined by MFI indices of stalk region-specific HM14 mAb varied among huFcµR WT and the seven N-terminal mutants (**Figure 3B**). By comparing with WT huFcµR, the receptor levels were comparable in three mutants (N66-, 79–83, and Y81C), clearly enhanced in another three mutants (E41Q, M42L, and 41–42; green columns), and significantly reduced in the 24–27 mutant (red column). Essentially the same conclusions were obtained with another HM7 mAb specific for an epitope near the ligand-binding site (**Figure 3C**). Notably, both N66- and 79–83 mutants, despite their comparable levels of surface FcµR, exhibited significantly diminished IgM ligand binding as compared with WT, suggesting that both N66 and K79-R83 are responsible for the constitutive IgM binding of human FcµR (**Figure 3D** red columns). Instead of five aa replacement (K79-R83), a single replacement mutant (Y81C) exhibited a comparable IgM binding as WT FcµR. The IgM binding results were essentially the same irrespective of the use of IgM ligands of human or mouse origin.

In the second series of experiments (n = 7), the GFP MFI of N109K mutant was significantly higher than WT and others (**Figure 4A** green column). Both WT and N109K mutant huFcµR expressed comparable levels of cell surface FcµR as determined by the HM14 mAb (**Figure 4B**), whereas the IgM binding activity with either mouse or human IgM was significantly reduced in the N109K mutant as compared with WT, suggesting an involvement of N109 in IgM binding of human FcµR (**Figure 4D** red column). In support of this, the MFI index of the ligand-specific HM7 mAb, unlike the stalk region-specific HM14 mAb, was significantly diminished in the N109K mutant compared with WT (**Figure 4C** red column). This result also implies that an epitope of human FcµR recognized by HM7 mAb is strongly susceptible to a point mutation of N to K at position 109. Collectively, the results from these mutational analyses suggest that the N residues at both positions 66 and 109 in CDR2 and CDR3 respectively (see model below), and the stretch from K to R at positions of 79 to 83 in the DE loop of human FcµR, are responsible for its constitutive ligand-binding activity.

### Altered Surface Receptor Expression of Human FcµR Mutants With KVEG24-27QLNV, E41Q, M42L, or EM41-42QL

Unlike the four huFcµR mutants (N66-, 79-83, Y81C, and N109K) which did not affect surface receptor expression defined by the reactivity of HM14 mAb, the 24–27 mutant exhibited significant reduction of both the surface receptor expression and IgM-binding activity (**Figures 3B–D** red columns). This suggested that the four aa stretch of K24-G27 in the A strand of huFcµR could be critical for maintaining the proper structure of the receptor expressed on the plasma membrane. On the other hand, intriguingly, three CDR1 mutants E41Q, M42L, and EM41-42QL showed a significant increase in their surface receptor expression in order: 41–42 >> E41Q > M42L (**Figures 3B, C** green columns). This increase was observed with both HM14 and HM7 mAbs as well as with IgM-ligand binding using TEPC183 paraprotein (**Figure 3D** green columns), although the increase with M42L was not statistically significant. Human IgM, instead of mouse IgM, paraprotein yielded the same results, thereby ruling out the possibility that such enhancement was due to the use of this particular TEPC183 IgM paraprotein. The finding of enhancement of both FcµR expression and ligand binding activity by replacing human E41 and M42 residues in CDR1 with mouse residues Q and L, respectively, was unexpected. However, when considering design of therapeutic interventions targeting FcµR in humans, this finding may turn out to be serendipitous.

### Modeling of Human and Mouse FcµR and Analysis of Stability

The melting temperatures (Tm) measured from molecular dynamics simulations using two different criteria, namely loss of native contacts and increase in R_g_, for all Ig-like domains of human FcµR of WT and mutant and mouse FcµR were in all but one case within one S.D. of each other (**Table 1**). Thus, none of the substitutions or the deletion causes a statistically significant difference in Tm (*i.e.*, stability) for human FcµR. However, the mouse FcµR domain shows a marginally significant lower Tm than human FcµR (*p* = 0.05).

**Table 1:** Predicted melting temperatures for FcµR lg-like domains of human WT or mutant and of mouse WT.

FcµR:	human									mouse
Type:	WT	24–27	E41Q	M42L	41–42	N66-	79–83	Y81C	N109K	WT
Criterion for melting temperature	Temperature K (C)									
<80% native contacts retained	336 (63)	342 (69)				330 (57)	336 (63)		333 (60)	327 (54)
Standard Deviation	5.7	12.2				11.9	5.1		9.6	5.8
Temperature at which Rg exceeds 14 Å	344 (71)	336 (63)				342 (70)	351 (78)		343 (70)	329 (56)
Standard Deviation	5.9	11.5				8.6	4.9		9.0	8.7

**Figure 5:**
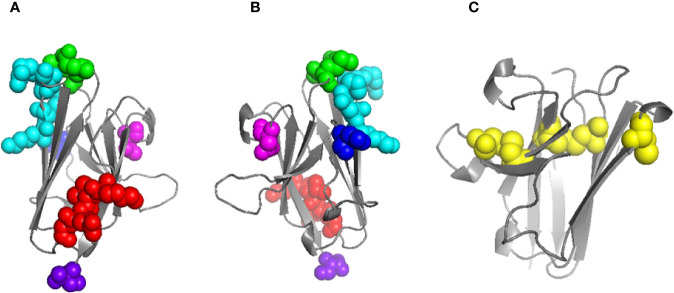
Models of human and mouse FcµR Ig-like domains. Model of human FcµR domain **(A)** and horizontally rotated by 180° **(B)** indicating the residues mutated in this study: K24-G27 (red), E41/M42 (green), N66 (blue), K79-R83 (cyan), N109 (magenta); the C-terminus is also indicated (purple). Model of mouse FcµR domain **(C)** showing all five C residues; four form two disulphide bridges (left and center) and the single free C residue at position 81 is shown exposed on the right. Models were generated using Swiss-Model (18).

Homology models were generated using pIgR D1 as a template for both human and mouse WT FcµR Ig-like domains, and then the substitutions of mouse aa sequences as described above, including the deletion at position 66 into the human sequence, were also modeled. None of the substitutions or the deletion caused significant changes to the main-chain conformation of the human FcµR domain, nor appeared to be incompatible in any way. The model and the locations of the substitutions and the deletion in human FcµR are shown in **Figures 5A, B**. **Figure 5C** shows the model of mouse FcµR and highlights the accessibility of the free cysteine residue, C81, not present in human FcµR.

### Modeling of the Complex Between Human FcµR and IgM

**Figure 6:**
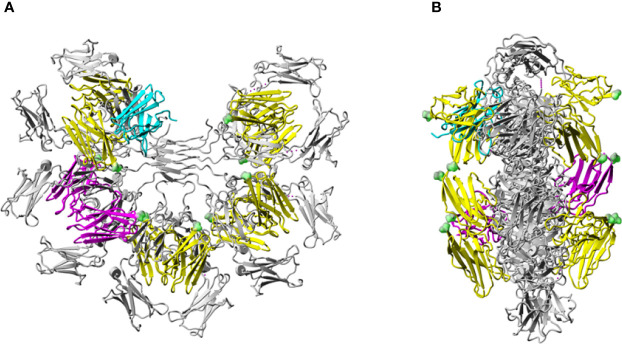
Model of the human FcµR/IgM interaction. Model of human FcµR Ig-like domains in complex with pentameric IgM-Fc **(A)** and rotated by 90° **(B)**. Pentameric IgM-Fc [PDB 6KXS, without J-chain or secretory component (SC)] (25) is shown in grey. The two FcµR domains docked onto heavy chains C and D of IgM-Fc are colored in magenta. Four other pairs of FcµR Ig-like domains located similarly on the four other Fc subunits of the IgM pentamer are colored in yellow. The location of the pIgR D1 (SC) (25) is shown in cyan. The C-termini of each of the 10 FcµR domains are indicated by green spheres.

**Figure 7:**
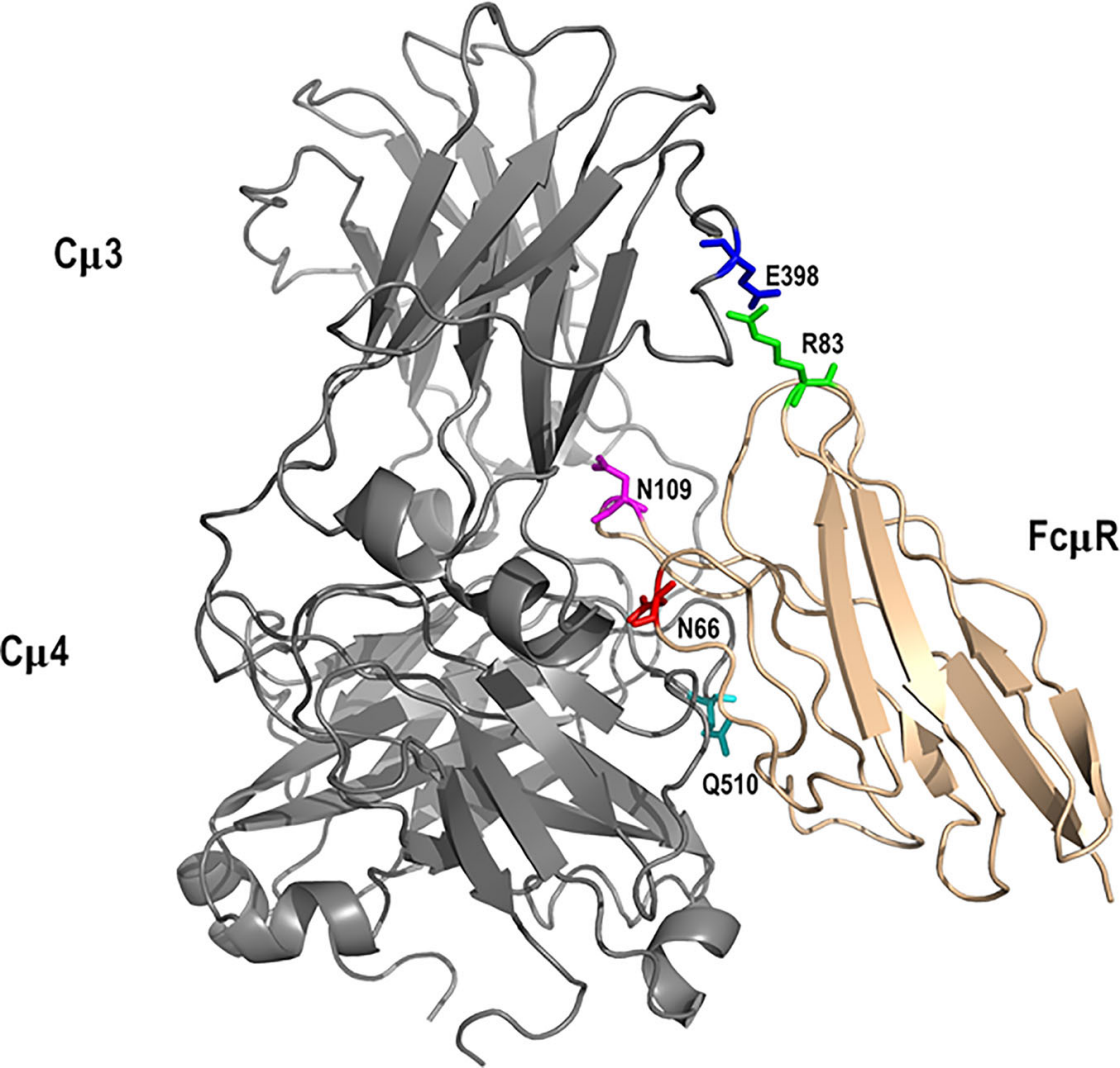
Interaction between FcµR and IgM-Fc subunit. Key residues in the modeled complex between the Ig-like domain of FcµR (tan) and an IgM-Fc subunit (grey) are indicated. They include N66 (red), R83 (green), and N109 (magenta) for FcµR and E398 (blue) and Q510 (cyan) for IgM-Fc. These residues, with the exception of E398, are based on our present and earlier experimental results (27).

The highest scoring docked model that satisfied the specified criteria described in Methods, including involvement of N66, K79-R83, and N109 of FcµR and Q510 of IgM-Fc chain C (27), had a score of 780. A similar but not identical mode of docking to IgM-Fc chain D (defining Q510 of IgM-Fc chain D as an interface residue) was found with a score of 924. These scores are consistent with the interaction affinity of K_a_ = 10^6^ M^-1^ (27), and we observe an approximately linear relationship between logK_a_ and the GalaxyTongDock score, based upon several test cases of known complexes mainly involving Ig-like domains, ranging from K_a_ = 10^4^ to 10^10^ M^-1^. The two docked FcµR domains are shown in magenta in **Figure 6**. Chimera-visualization system (31) was used to count the number of contacts to Cµ3 and Cµ4, establishing that over 75% were to Cµ4. A closer view of the interaction of one FcµR Ig-like domain with the C and D chains of IgM-Fc, with the key residues indicated, is shown in **Figure 7**, and the involvement of Cµ3 and Cµ4 domains, but principally the latter, can be seen. All four residues (N66, R83, and N109 of FcµR and Q510 of IgM-Fc) were clearly at the interface, suggesting their potential involvement in IgM binding and consistent with the experimental data. In addition to Q510, E398 in Cµ3 was also predicted by this model to contact R83 in the DE loop of FcµR. We did not see contact of E41/M42 in the CDR1 of FcµR with any part of IgM-Fc. By superposing chains C and D on each of the other IgM-Fc subunits of the pentamer (chains A and B, E and F, *etc.*), eight further FcµR domains were located (31), as shown in yellow in **Figure 6**. The modeling thus suggests that there are up to ten accessible binding sites for FcµR in each IgM-Fc (or IgM) pentamer. The C-termini of all the FcµR domains are exposed (indicated by green spheres in **Figure 6**) and suitably positioned for connection *via* the stalk region to the membrane. **Figure 6** also shows that one of the FcµR domains (located on chain A) overlaps slightly with pIgR D1 (25), shown in cyan; the mode of binding predicted for FcµR is clearly very different to that observed for pIgR D1, as suggested by (25). While this model satisfies the experimental data, it cannot be considered a unique solution.

## Discussion

The aim of the present study is to explore the molecular basis for differences in IgM binding observed in transductants stably expressing human and mouse FcµRs. Human receptor-bearing cells bound to IgM regardless of their cell growth phase (*constitutive* binding), whereas mouse receptor-bearing cells did so only in the pre-exponential growth phase (*transient* binding), although the cell surface levels of FcµR determined by receptor-specific mAbs did not significantly change. Subsequent domain swapping analysis revealed that this difference was directly attributed to the Ig-like domain rather than indirectly to other parts of the receptor (6). We hypothesized that the replacement of aa residues potentially involved in the ligand binding of human FcµR with the corresponding mouse residues might result in loss of its constitutive ligand binding activity. In the present mutational analysis, several remarkable features of the human FcµR were revealed. *First*, at least three sites of human FcµR, *i.e.*, two N residues N66 and N109 predicted to lie in the CDR2 and CDR3, respectively, and a stretch of five aa from K79 to R83 in the DE loop, were responsible for its constitutive ligand binding potential. *Second*, replacement of E41 and M42 in the CDR1 with the corresponding mouse residues Q and L resulted in enhancement of binding of both receptor-specific mAbs and of IgM-ligands. *Third*, the four aa stretch of K24 to G27 in the presumable A strand of human FcµR was critical for maintenance of its proper structure on the plasma membrane as the replacement mutant caused marked reduction of both reactivities with receptor-specific mAbs and IgM ligands. Results from computational modeling identified a possible mode of interaction between FcµR and IgM consistent with the involvement of loops including N66, R83 and N109 in human FcµR and E398 and Q510 of IgM-Fc, and demonstrated a different mode of IgM binding for FcµR compared with that of pIgR.

Substantial loss of IgM binding of human FcµR by replacing its N66, K79-R83, and N109 with the mouse equivalents suggested that these three sites were at least required for the constitutive IgM-binding of human FcµR. The loss of binding was not due to reduction of the cell surface expression of FcµR as determined by receptor-specific mAbs. Furthermore, none of the substitutions in the present studies caused significant alterations in the main conformation of the receptor as determined by modeling of the substitutions or changes in the melting temperature assessed by molecular dynamics simulations (**Table 1**). These residues were respectively located in the CDR2, DE loop, and CDR3 regions of human FcµR (**Figures 5** and **S3**) based on the pIgR D1 (PDB 5D4K) as a template. Like pIgR and Fcα/µR, the FcµR CDR2 loop is also very short with only two residues in many mammals including humans, but missing one residue in rodents (2). It is thus conceivable that removal of one of the two residues (N66) from human FcµR CDR2 (N66-) may profoundly affect its IgM binding activity. In this regard, the CDR2 in rabbit pIgR D1, like mouse FcµR, is also missing one residue and lacks IgM binding; replacement of missing aa with the human equivalent gains IgM binding and removal of the corresponding residue in human pIgR CDR2 results in substantial loss of IgM binding (34).

The docked model of FcµR and IgM-Fc pentamer proposed in **Figure 7** clearly demonstrates that three loops including N66, R83, and N109 of FcµR as well as the loops containing Q510 in Cµ4 and E398 in Cµ3 of IgM are all at the interface, suggesting their potential interactions. This FcµR/IgM complex model is thus consistent with substantial loss of IgM binding in mutants by substituting K79-R83 or N109 with the mouse equivalents, and the involvement of both Cµ4 (Q510) and Cµ3 (E398) domains is also consistent with previous experimental data (2, 27, 29). The finding that R83, the C-terminal residue of the above five aa stretch in the DE loop, interacted with E398, was unexpected. It has been shown that the position of the CDR3 in pIgR D1, unlike Ig VH and VL domains, is tilted away from the ABED sheet and the other CDRs (33). Furthermore, the pIgR CDR3 loop is stabilized by hydrogen bonds between N115 within the loop and two residues (R52 in the C strand and T66 in the C’ strand) (33). These characteristics were also preserved in human FcµR based on computational modeling (see **Figures 5** and **S3**).

Among the three known IgM binding receptors (FcµR, pIgR, and Fcα/µR), many features are conserved in their ligand-binding domains, such as two intra-chain disulfide bonds of C37-C104 and C49-C58 and a salt bridge between R75 and D98 in human FcµR (6, 33). The greatest difference between FcµR and the other two receptors is in the CDR1 region. The CDR1 of pIgR and Fcα/µR consists of nine aa, whereas the corresponding region of FcµR consists of only five aa (2) (see **Figure S3**). Furthermore, the R49 of human pIgR, which is solvent exposed and thought to directly interact with polymeric IgA, is replaced by a non-charged residue of M or L at position 42 in human and mouse FcµR, respectively. These differences could account for the stringent ligand specificity of FcµR for IgM only, but not for polymeric IgA and IgM like the other two receptors. The modeled structure of human FcµR showed that E41 and M42 in the CDR1 as well as N66 in the CDR2 and K79-R83 in the DE loop were close to one another in three-dimensional space (**Figure 5**). We initially predicted that human FcµR CDR1 mutants (E41Q, M42L, or EM41-42QL) might also profoundly modulate their IgM ligand binding. Intriguingly, the CDR1 mutants enhanced both receptor expression and IgM binding activity. The molecular basis for this enhancement remains to be elucidated, but several possibilities such as post-translational modifications involved glutamine residues or indirect influence on its conformation may be considered. Alternatively, EM41-42QL mutant simply enhances the cell surface expression of FcµR without affecting IgM-binding affinity. Whatever the mechanisms for enhancement of IgM binding by human FcµR EM41-42QL mutant, this would be a serendipitous finding when considering the potential clinical applications of FcµR. In a mouse model of myelin oligodendrocyte glycoprotein (MOG)-induced experimental allergic encephalomyelitis (EAE), administration of a recombinant soluble human FcµR/IgG fusion protein into EAE-susceptible C57BL/6 mice resulted in delaying or ameliorating their disease depending on the time points of injection (5). Since IgM anti-MOG antibody also participates in the demyelination in EAE, such soluble FcµR/IgG may thus act as a decoy receptor. If this is indeed the case and the MOG-induced EAE resembles human multiple sclerosis (MS) in pathogenesis, then the FcµR EM41-42QL mutant offers an intervention in individuals with MS, using gene editing that permits reliable introduction of point mutations in induced pluripotent human stem cells (35). Another as yet uninterpretable observation was marked loss of both receptor expression and IgM binding activity in the human FcµR mutant of KVEG24-27QLNV, because the Tm values of WT and this N-terminal mutant measured from molecular dynamic simulations were all comparable (**Table 1**).

There are many precedents of receptors with distinctive ligand binding properties among different species, and this issue is important especially when considered their clinical applications. Examples related to immunological fields include the binding of HIV gp120 or EBV gp350/220 to human *vs* mouse CD4 or CD21, respectively (36), distinct differences in structural requirements for the interactions between human and mouse albumin with their respective neonatal Fc receptors (37), and structure-function differences in different 4-hydroxipiperidine CCR1 antagonists for human *vs* mouse CCR1 (38). For the interaction of HIV gp120 and CD4, the murine CD4 ectodomain has an overall 50% identity with the human counterpart at the aa level but fails to bind HIV gp120. Clayton *et al.* took advantage of this species difference and replaced non-conserved human residues with the corresponding mouse ones, thereby identifying several aa residues critical for HIV gp120 binding in human CD4 molecules (36). We took a similar approach and identified three critical sites (N66 in CDR2, K79-R83 in DE loop, N109 in CDR3) for constitutive IgM binding of human FcµR, and the results were confirmed by structural modeling analyses.

Regarding IgM, recent technical advances in single-particle negative-stain electron microscopy (EM) (39) and cryo-EM (25) have resulted in dramatic advances in our understanding of its structure. According to the textbook model, IgM is a symmetric pentamer like a planar star-shape pentagon with the Fab fragments pointing away from the inner core of the Fc regions (40). By contrast, according to the recent EM structures, IgM is an asymmetric pentamer, resembling a hexagon with a missing triangular segment where several proteins fit, such as the J chain, the pIgR D1 (secretory component) and the apoptosis inhibitor of macrophages (AIM) (25, 39). AIM [also called CD5-like antigen or soluble protein α (Spα)] is a glycoprotein of ~45 kDa secreted by macrophages, which facilitates repair during different diseases and was originally identified as an IgM-binding protein (41). Unlike these IgM-binding proteins, our docking model suggests that FcµR does not fit in this “gap” region, but instead, interacts with the Cµ4 domains, and to a lesser extent the Cµ3 domains, of each monomeric subunit of the human IgM pentamer (**Figure 6**). Furthermore, and consistent with this model, FcµR, unlike pIgR, can interact with both J chain-containing pentameric and J chain-lacking hexameric IgM with similar affinities (9) and lacks two thirds of the critical residues of human pIgR in interacting with IgM [*i.e.*, V47, N48, (H50, R52), Y73 and L119; see **Figure S3**] (25). Thus, these findings all suggest that FcµR binds IgM in a different fashion compared with pIgR. **Figure 6** also shows that with every subunit of IgM-Fc harboring a binding site for FcµR on each side of the IgM-Fc “disc”, a single IgM molecule could be bound by more than one FcµR at the same time.

It is likely that the transient IgM binding observed with mouse, but not human, FcµR-bearing cells during culture results from posttranslational modifications. In the case of FcγRs, it is now evident that carbohydrate moieties on the inhibitory and activating forms of the receptor, as well as on their IgG ligands, are strongly associated with the respective receptor functions (1). In FcµRs of both human and mouse, one third of the mature receptor molecular mass is made up of O-linked, but no N-linked, glycans (2, 16, 42). While the role of carbohydrates on mouse FcµR in its IgM binding is unknown, recent studies of the human receptor showed that its IgM-ligand binding and subsequent receptor-mediated internalization occur irrespective of its glycosylation (29). Since mouse FcµR has an additional solvent-exposed free C residue at position 80 (Figs. 5C and S1), this residue may form an inter-chain disulfide bond, and the resultant homo- or hetero-dimeric form of the receptor may overcome the disadvantage of having only one residue in the CDR2, resulting in a transient IgM binding. Alternatively, we note the recent findings that “labile” disulfide bonds are commonly present in cell surface proteins, and that such “labile” disulfide bonds are involved in regulating molecular functions of the constituent C residues *in vivo* (43). In this regard, C80 may participate in a “labile” disulfide bond in FcµR (*i.e.*, C49-C58), resulting in a conformational change of the receptor with an IgM-binding activity. Introduction of this C residue into the corresponding human site (Y81C mutant) did not enhance IgM binding. During B-lineage differentiation in mice, the cell surface expression of FcµR is detectable from bone marrow immature B cells to plasmablasts, except for a transient down-modulation during germinal center reactions (16, 44). What posttranslational modifications other than O-glycosylation that the mouse FcµR receives *in vivo* to gain its ligand binding activity need to be clarified. In this regard, ubiquitously expressed HLA heavy chain molecules have been shown to receive a unique tyrosine-sulfonation selectively on the surface of memory B and plasma cell populations (45). This particular modification was identified by using a lamprey-derived monoclonal antibody, suggesting a distinct structural difference between Y-sulfated and -non-sulfated HLA I heavy chain molecules that is efficiently recognized by a leucine-rich lamprey antibody, reminiscent of the recognition of pathogen-associated molecular patterns by leucine-rich Toll-like receptors (46).

Collectively, by taking advantage of the difference in IgM binding of human and mouse FcµRs, we identified three critical residues in IgM binding of human FcµR: N66 in the CDR2, R83 in the DE loop and N109 in the CDR3. A proposed mode of binding to IgM differs from that of pIgR and involves contact with domains of Cµ4 (including Q510) and Cµ3 (including E398). Serendipitously, we found that substitution of E41/M42 in the CDR1 of human FcµR with the mouse equivalents Q/L enhances both receptor expression and IgM binding potential. We can thus now suppress or enhance FcµR binding to IgM by recent advances in gene editing that permit reliable introduction of point mutations in induced pluripotent human stem cells (35). These findings would help in future development of preventive and therapeutic interventions targeting FcµR.

## Data Availability Statement

The raw data supporting the conclusions of this article will be made available by the authors, without undue reservation.

## Author Contributions

HK, CS, and KA-Q designed the mutations and made stable transductants (**Figures 1**, **S1**, and **S3**). CS and KA-Q conducted flow cytometric and statistical analyses (**Figures 2** **–** **4** and **S2**). RC and BS performed computational structural analysis (**Figures 5** **–** **7** and **Table 1**). PE and AR intellectually contributed. RC, BS, and HK wrote the paper. All authors contributed to the article and approved the submitted version.

## Funding

This study was supported by the Deutsches Rheuma-Forschungszentrum institutional funds to HK and BBSRC Project Grant (BB/K006142/1) to BS.

## Conflict of Interest

The authors declare that the research was conducted in the absence of any commercial or financial relationships that could be construed as a potential conflict of interest.

## Supplementary Material

Supplementary Figure 1 Amino acid sequence alignment of human and mouse FcµR. Amino acid sequences (single-letter code) of human (Hu) and mouse (Mo) FcµR are aligned with the aa position from the first M residue in top and bottom, respectively. Amino acid identity is indicated as dots (•) and a deletion by dashes (-). Missing or additional regions are highlighted in yellow. Red vertical lines indicate the exon boundaries: signal peptide (*Sp*), Ig-like domain (*Ig-D*), stalk region 1 and 2 (*St-1, St-2*), transmembrane segment (*TM*), and cytoplasmic tail 1, 2 and 3 (*Cy-1, Cy-2, Cy-3*).

Supplementary Figure 2 MFI indices of mouse FcµR-specific mAbs, MM3 and MM24, in each transductant. MFI indices of the reactivity of MM3 and MM24 mAbs with the indicated transductants were plotted as mean ± 1 SD from three (*top panel*) and seven (*bottom panel*) experiments. Lines correspond with the MFI index of human FcµR WT. ***P* < 0.01, ****P* < 0.001 when compared with the human FcµR WT transductant.

Supplementary Figure 3 Amino acid sequence alignment of IgM binding receptors. The Ig-binding domains of FcµR, Fcα/µR and pIgR from human (hu) and mouse (mo) were aligned using the Clusal Omega multiple alignment program (EMBL-EBI). Amino acid (aa) identity is indicated as dots (•), gap as blank, and a deletion by dashes (-). Residues conserved in all three receptors and Fcα/µR and pIgR are highlighted in yellow and gray, respectively. Substituted residues in the present studies are highlighted in blue. The numbers indicated at the top and bottom correspond with the aa position from the Met residue of human FcµR and pIgR, respectively. The positions of each ß strand (black arrows) and CDRs (red lines) of human pIgR (33) are indicated. Accession numbers of the sequences other than FcµRs are: Fcα/µR of human (AAL51154) and mouse (NP_659209); pIgR of mouse (AAC53585) and human (EAW93516).
