# Supplementary figures and images for: Identification of Amino Acid Residues in Human IgM Fc Receptor (FcµR) Critical for IgM Binding
Source: Front Immunol. 2021 Jan 27;11:618327. doi: 10.3389/fimmu.2020.618327 (PMC7873564; doi:10.3389/fimmu.2020.618327)

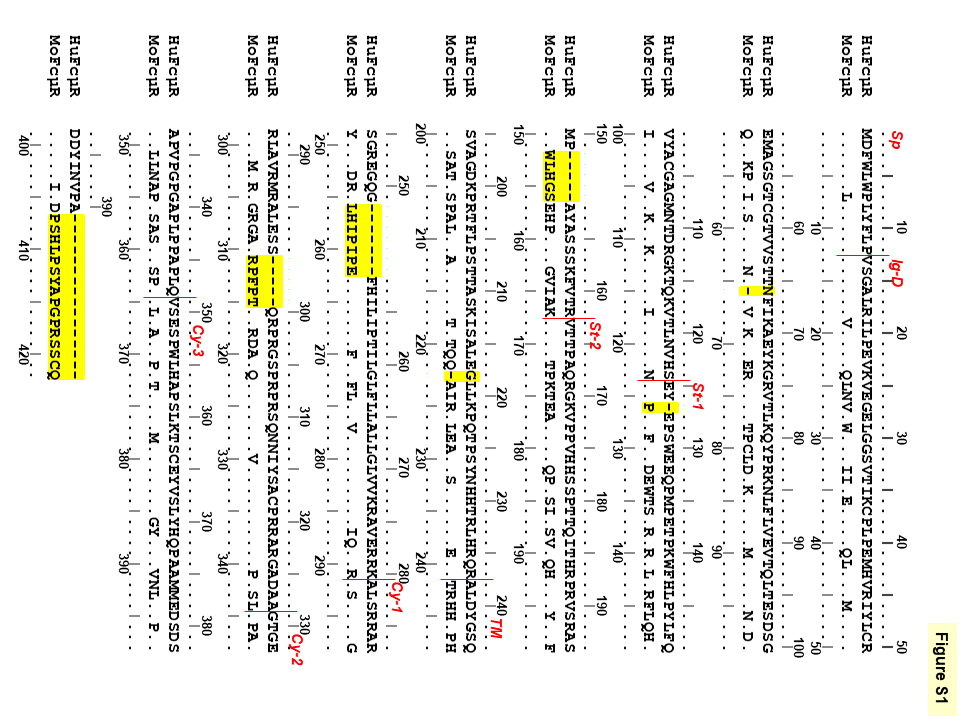

Supplement: Supplementary Figure 1 — Amino acid sequence alignment of human and mouse FcµR. Amino acid sequences (single-letter code) of human (Hu) and mouse (Mo) FcµR are aligned with the aa position from the first M residue in top and bottom, respectively. Amino acid identity is indicated as dots (•) and a deletion by dashes (-). Missing or additional regions are highlighted in yellow. Red vertical lines indicate the exon boundaries: signal peptide (Sp), Ig-like domain (Ig-D), stalk region 1 and 2 (St-1, St-2), transmembrane segment (TM), and cytoplasmic tail 1, 2 and 3 (Cy-1, Cy-2, Cy-3). [file Image_1.tif]

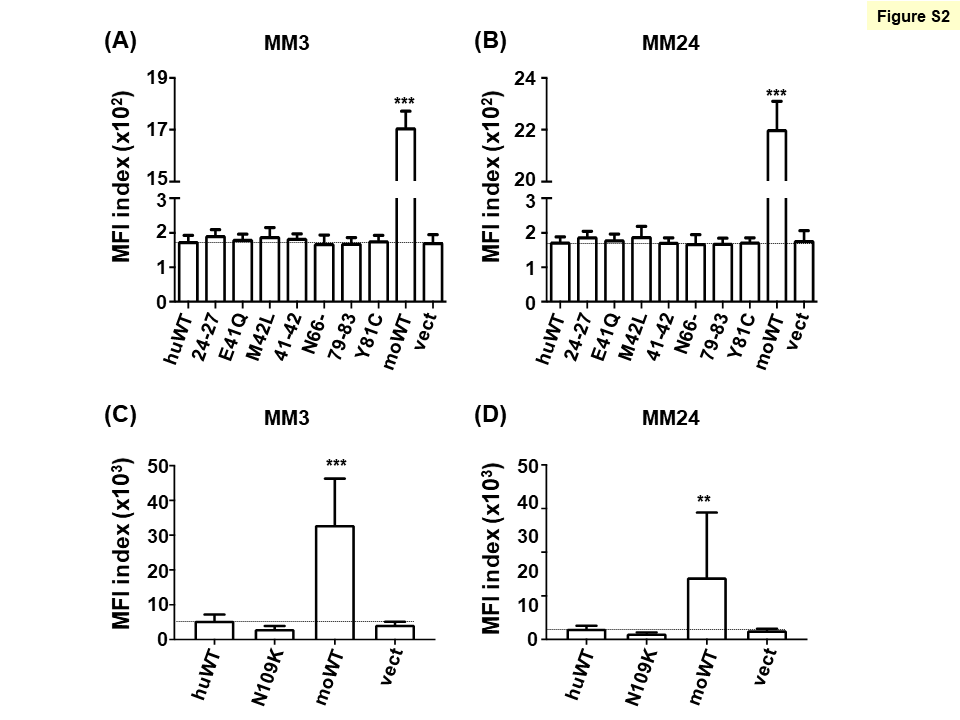

Supplement: Supplementary Figure 2 — MFI indices of mouse FcµR-specific mAbs, MM3 and MM24, in each transductant. MFI indices of the reactivity of MM3 and MM24 mAbs with the indicated transductants were plotted as mean ± 1 SD from three (top panel) and seven (bottom panel) experiments. Lines correspond with the MFI index of human FcµR WT. **P < 0.01, ***P < 0.001 when compared with the human FcµR WT transductant. [file Image_2.tif]

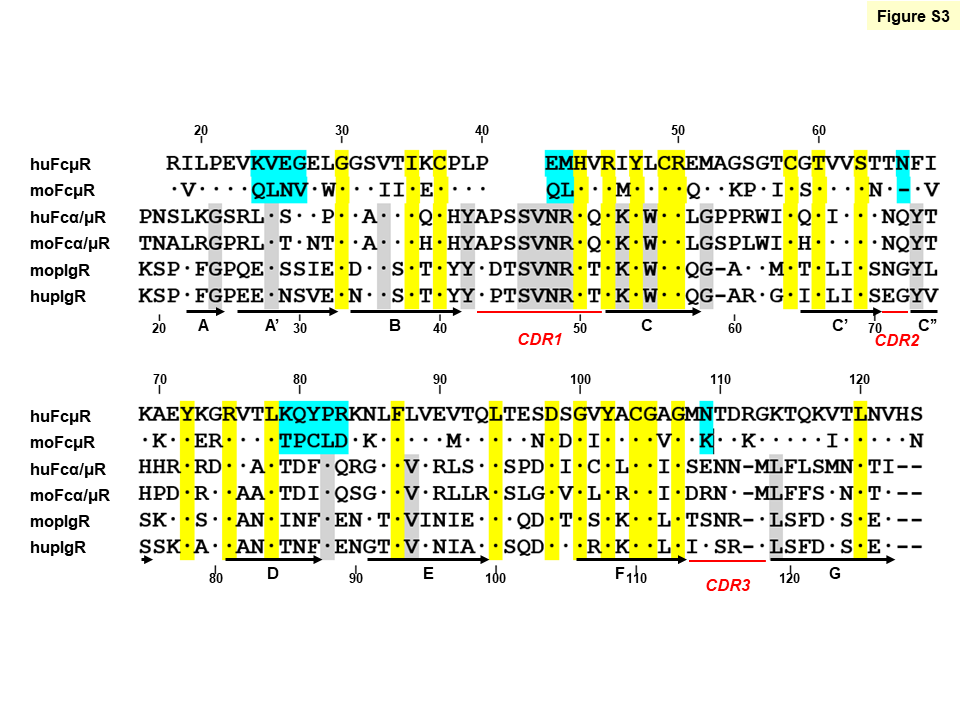

Supplement: Supplementary Figure 3 — Amino acid sequence alignment of IgM binding receptors. The Ig-binding domains of FcµR, Fcα/µR and pIgR from human (hu) and mouse (mo) were aligned using the Clusal Omega multiple alignment program (EMBL-EBI). Amino acid (aa) identity is indicated as dots (•), gap as blank, and a deletion by dashes (-). Residues conserved in all three receptors and Fcα/µR and pIgR are highlighted in yellow and gray, respectively. Substituted residues in the present studies are highlighted in blue. The numbers indicated at the top and bottom correspond with the aa position from the Met residue of human FcµR and pIgR, respectively. The positions of each ß strand (black arrows) and CDRs (red lines) of human pIgR (33) are indicated. Accession numbers of the sequences other than FcµRs are: Fcα/µR of human (AAL51154) and mouse (NP_659209); pIgR of mouse (AAC53585) and human (EAW93516). [file Image_3.tif]
